# Suggesting Indicators of Age-Friendly City: Social Participation and Happiness, an Ecological Study from the JAGES

**DOI:** 10.3390/ijerph19095096

**Published:** 2022-04-22

**Authors:** Kazushige Ide, Seungwon Jeong, Taishi Tsuji, Ryota Watanabe, Yasuhiro Miyaguni, Hirotaka Nakamura, Miyako Kimura, Katsunori Kondo

**Affiliations:** 1 Center for Preventive Medical Sciences, Chiba University, Yayoi-cho, Inage, Chiba 263-8522, Chiba, Japan; tsuji.taishi.gn@u.tsukuba.ac.jp (T.T.); ryota.watanabe.1222@gmail.com (R.W.); kkondo@chiba-u.jp (K.K.); 2 Department of Community General Support, Hasegawa Hospital, Yachimata 289-1113, Chiba, Japan; 3 Department of Community Welfare, Faculty of Health Sciences, Niimi University, Nishigata, Niimi 718-8585, Okayama, Japan; k-jeong@niimi-u.ac.jp; 4 Center for Gerontology and Social Science, Research Institute, National Center for Geriatrics and Gerontology, 7-430 Morioka-cho, Obu 474-8511, Aichi, Japan; miyaguni@n-fukushi.ac.jp; 5 Faculty of Health and Sport Sciences, University of Tsukuba, 3-29-1 Otsuka, Bunkyo 112-0012, Tokyo, Japan; 6 Faculty of Social Welfare, Nihon Fukushi University, 35-6 Okudaegemae, Mihama-cho, Chita-gun 470-3295, Aichi, Japan; 7 Department of Nursing, Asahi University, 1851 Hozumi, Mizuho 501-0296, Gifu, Japan; nakamura@alice.asahi-u.ac.jp; 8 Department of Preventive Medicine, St. Marianna University School of Medicine, 2-16-1, Sugao, Miyamae, Kawasaki 216-8511, Kanagawa, Japan; mkimura@marianna-u.ac.jp

**Keywords:** older people, healthy aging, age-friendly community, community diagnostic indicator, happiness

## Abstract

Ascertaining progress in building age-friendly cities (AFCs) requires community diagnostic indicators. This study examines the relationship between social participation and happiness at the municipal level. The data from the Japan Gerontological Evaluation Study (JAGES) from 2013, 2016, and 2019, comprising 442,079 older people from 289 municipalities, are used. We also employ linear mixed-effects models to evaluate the association between social participation and happiness. In these models, we adjust for seven variables as potential confounders. This study reveals that the higher the social participation, except for neighborhood association, the higher the state of happiness (*B* = 0.14–0.30). Our study suggests that social participation is useful, as a community diagnostic indicator, for monitoring the progress of building AFCs, developing strategies, and creating evidence.

## 1. Introduction

Because of a rapidly aging global population, all aspects of society will most likely be affected. Thus, to support older people’s aging in place, urban planners and local governments need to provide an age-friendly environment at the community and city levels so that this demographic’s daily needs can be fully met [1,2]. Research and social implementation have been underway in various fields to build a healthy and long-living society. For example, the World Health Organization (WHO) proposed age-friendly cities (AFCs) [3] in 2007, with a focus on creating livable communities for older people. In 2010, the Global Network of Age-friendly Cities and Communities (AFCC) was launched to promote the implementation of policy recommendations [4]. Consequently, the growth of the AFCC has generated numerous research publications [5]. In the process of building AFCs, it is crucial to monitor, evaluate, measure, and assess the age-friendliness of cities and communities [6]. Demonstrating the success of public policy initiatives on the broad scale that characterizes age-friendly communities requires adequate measurement instruments [7].

Several studies have been conducted on AFC measurement instruments, which are also called community diagnostic indicators [4,6,7,8,9,10,11,12,13,14,15]. These studies were based on eight interconnected domains of life (i.e., community and healthcare, transportation, housing, social participation, outdoor spaces and buildings, respect and social inclusion, civic participation and employment, and communication and information) by the WHO that underlie its AFC concept to diagnose and assess the physical and social environment [16]. These studies involved the development of measurement instruments, such as the Age-Friendly Environment Assessment Tool (AFEAT) [8] and the Age-Friendly City and Community Questionnaire (AFCCQ) [4,6], and the use of publicly available data or original surveys [7,9,10,11,12,13,14,15].

In the AFC framework, health and well-being are defined as impact indicators [16]. It is hoped that impact indicators will come about (at least in part) through age-friendly improvements in physical and social environments [16]. Previously, studies focused on the negative aspects of health; however, in recent years, based on the perspective of leading and realizing a good life, various concepts that emphasize the positive aspects of health—such as sustainable well-being, including happiness and positive psychology—have received social and academic attention [17,18]. A previous study also reported that countries with high levels of well-being exhibit longer life expectancy, suggesting that governments should foster well-being to support long-living populations [19]. Additionally, health status, financial satisfaction, and freedom of choice have been reported in meta-analyses as factors associated with individual-level well-being [20,21].

In Japan, age-friendly policies include the implementation of community-based integrated care wherein medical, long-term, and preventive care are integrated into communities where older people reside [22]. Based on such policies, local governments implement social innovations, such as group movement, and other social and cultural activities [22,23]. Several previous studies [24,25,26,27] reported that the social participation of older adults is protective against functional disability and dementia onset. In previous studies [24,25,26,27], the establishment of social interaction and the maintenance of physical and cognitive functions through social participation inhibited functional disability and dementia onset. Therefore, from the eight domains of AFC, this study only focuses on social participation, which is often emphasized as an intervenable target in public health policies and an important component of healthy aging [28]. Based on previous studies, social participation [29,30,31,32] and altruistic social activities [33] are associated with individual-level well-being.

Thus far, many studies have examined factors related to well-being at the individual level, including social participation [20,21,29,30,31,32,33]. The associations at the individual level do not always correspond to those at the ecological level. Therefore, it is necessary to verify the relationship at the ecological level, if social participation and well-being are to be used as community-level indicators to manage the progress of building AFCs. Several previous studies [4,10,11,14,34,35] examined disparities in social participation and well-being and their association at the national, city, and community levels. However, few reports have examined each type of social participation. To determine the relationship between social participation and well-being at the ecological level, we examined it at the municipal level. In most countries, municipalities are the smallest administrative units that implement administrative plans, such as health and welfare initiatives, for older adults. Like other countries, municipalities in Japan have widely different social backgrounds and sociodemographic characteristics [36].

Considering social participation and well-being for building AFCs, the data from the Survey of Needs [37] could be utilized in Japan. This particular survey, conducted by the Ministry of Health, Labour and Welfare (MHLW), included the models implemented by numerous municipalities that obtained basic information to create long-term care insurance programs [37]. The AFC concept does not mention the specific indicators of well-being that should be used [3,16]. Thus, we focused on the availability indicator of happiness, a type of psychological well-being [38] that is a basic item in the Survey of Needs [37]. In community surveys and cross-cultural comparisons, using a single question to measure happiness has been demonstrated to be reliable, valid, and feasible [39,40]. Moreover, reliability, validity, and feasibility are important properties required for community diagnostic indicators in AFCs. Monitoring happiness can provide valuable information about demographic trends and might be useful in gauging the impact of public policies [41].

In summary, this study aims to examine the relationship between social participation by type and happiness at the municipal level.

## 2. Materials and Methods

### 2.1. Study Participants

In this study, we used repeated cross-sectional data from three waves (2013, 2016, and 2019) from the Japan Gerontological Evaluation Study (JAGES). The JAGES is one of the few population-based gerontological studies that focus on the social determinants of health and the social environment among functionally independent older adults aged 65 years and older in Japan [42,43]. In Japan, municipalities are the smallest administrative units, followed by village, town, and city, in terms of size. The ward is a subordinate body of an ordinance-designated city with a population of 500,000 or more. In each wave, 78, 92, and 122 municipalities (village, town, city, and ward) participated, respectively. The total population of the participating municipalities ranged from 1419 (Katsurao Village) to 3,745,796 (Yokohama City) [44]. Yokohama City consists of 18 wards, with the smallest population of 101,770 in Nishi Ward and the largest population of 308,354 in Aoba Ward [44]. In large municipalities, random sampling was used, while in the small and medium-sized municipalities, a census of the residents was conducted. Additionally, in the large municipalities, the participants who completed the questionnaire during the previous survey were oversampled in the subsequent survey. In the three waves, questionnaires were collected from 137,756, 196,438, and 253,678 participants, with response rates of 71.1%, 71.1%, and 70.2%, respectively. Further, 165,503 responses were excluded from our analysis for the following reasons: (i) incorrect answers for sex and age; (ii) missing addresses or activities of daily living; (iii) having self-reported physical or cognitive decline; and (iv) one municipality whose income was not included in the survey. The final number of participants was 442,079 in 289 municipalities (i.e., 2013: 119,410 from 77 municipalities; 2016: 156,143 from 91 municipalities; and 2019: 167,526 from 121 municipalities). The actual number of participating municipalities, excluding the multiple participants, was 133. Among these, 47 municipalities participated in only one wave, 16 participated in two waves, and 70 participated in all three waves.

This study was reviewed and approved by the Ethics Committees at Nihon Fukushi University, the National Center for Geriatrics and Gerontology, and Chiba University. This study was also performed by the principles of the Declaration of Helsinki. In the 2013 and 2016 surveys, all participants were informed that participation in the study was voluntary and that completing the questionnaire and returning it by mail was an indication of consent. For the 2019 survey, a checkbox for consent was placed on the questionnaire because of the revised ethical guidelines.

### 2.2. Dependent Variable

We used the data in which responses were aggregated at the municipal level. The dependent variable was happiness, which is one type of psychological well-being [45,46,47,48,49,50]. Happiness has been sometimes used interchangeably with “well-being” and “quality of life” [38]. Therefore, we used happiness, which is a part of psychological well-being; happiness is also included in the Survey of Needs [37]. In the Survey of Needs, well-being is measured by the following questions: “To what degree do you feel that you are currently happy?” Responses to this question were based on a scale from 0 (very unhappy) to 10 (very happy). Only the 2013 wave included a different scale: from 1 (very unhappy) to 10 (very happy). This difference was because of a change in the template of the Survey of Needs [37]. Overall, measuring happiness with this single item has been reported to be reliable, valid, and feasible in community surveys and cross-cultural comparisons [39,40].

Consistent with previous studies [33,51] that examined the relationship between social participation and happiness, participants with a score of 8 or higher were defined as “having a sense of happiness”. However, research on the cutoff point for happiness as a community diagnostic indicator remains insufficient. Therefore, for sensitivity analysis, we conducted an analysis wherein the cutoff point for happiness was set at 7 or more [52] and 5 or fewer points.

### 2.3. Independent Variable

The independent variable was social participation. Consistent with the AFC framework [16], we defined social participation as the participation of older people in community organizations. We used the following seven types of social participation, which were included in all three waves: volunteer groups (volunteering), sports groups or clubs (sports), hobbies groups (hobbies), senior citizen clubs (seniors), neighborhood associations (neighborhood), learning or cultural groups (learning), and activities to teach skills or pass on experiences to others (skills). These aspects were included in the Survey of Needs template and used in questions to obtain the social participation status of older people in Japan [37]. We measured social participation with the following question: “How often do you participate in the following clubs or groups?” The participants were given the following choices: “Almost every day”, “Twice or thrice a week”, “Once a week”, “Once or twice a month”, “A few times a year”, and “Never”. The responses were categorized as “yes” if participants selected options from “Almost every day” to “Once or twice a month” and “no” if they selected “A few times a year” and “Never”. This classification was based on previous studies on community diagnostic indicators [53,54,55] and several types of community diagnosing tools such as the WHO’s Urban Health Equity Assessment and Response Tool (Urban HEART) [56] and the MHLW’s Community-Based Integrated Care: Visualization System [57].

### 2.4. Covariates

Concerning previous studies related to well-being [19,20,21,52,58,59] and social participation [29,30,31,32,33,53,54,55], we used the following municipal level aggregates: low equivalent income (less than 2 million); low education (less than 10 years); living alone; self-reported medical illness; poor self-rated health; and depression (Geriatric Depression Scale-15: 5 points or more). Furthermore, we calculated the population density of habitable land by dividing the population by the area of habitable land at the municipality level (based on national statistical data) [44,60]. Thereafter, the municipalities were classified into the following three categories according to the calculated population density of habitable land: metropolitan (≥4000 people/km^2^), urban (1000–3999 people/km^2^), and rural (<1000 people/km^2^) [61].

### 2.5. Statistical Analysis

A simple comparison of the values aggregated at the municipal level is subject to differences in terms of the age structure within each municipality [53,54,55]. To compare the various indicators among municipalities with different age structures, the survey applied the age standardization method used by the MHLW and adjusted all the variables for age [62,63]; thus, this enabled us to more accurately compare the regions without focusing on the different age compositions in the groups. Based on the recommendation of the MHLW, the smoothed population data from 2015 were used as the reference population [63].

Considering the hierarchical structure of pooled data for three waves, we used linear mixed-effects models to evaluate the association between social participation and well-being at the municipal level. The data consisted of an iterative cross-sectional design that had two levels wherein Level 1 municipal level aggregates were nested within Level 2 survey years. We also utilized the forced imputation method to calculate the unstandardized coefficients (*B*), along with 95% confidence intervals (CIs) and *p*-values. In each model, we individually introduced the seven types of social participation.

In Model 1, to remove the influence of potential confounding factors, we adjusted the aggregate values for low equivalent income, low education, living alone, living alone at the municipal level, and population density of habitable land. In Model 2, we adjusted for all of the factors as in Model 1, including the aggregate values for self-reported medical illness, poor self-rated health, and depression. Because these three aspects can be taken as both confounders and intermediate factors, they were separately entered in Model 2. For all of the statistical analyses, Stata 17/IC software was used (StataCorp, College Station, TX, USA).

## 3. Results

Table 1 presents the municipal level aggregate values for each variable, after adjusting for age. The mean percentage of the 289 municipalities with a score of 8 or higher for happiness was 51.4% (standard deviation (SD), 3.7), while the mean percentages of the 289 municipalities with a score of 7 or higher and 5 or lower for happiness were 69.1% (SD, 4.0) and 20.2% (SD, 3.7), respectively. These findings reveal the disparities among the municipalities at all cutoff points in the age-adjusted, municipal level aggregated values of happiness.

Using the results of the most recent 2019 survey as an example, we described the differences among the municipalities in the aggregate level of happiness. Specifically, in the 2019 survey, there was a 19.2%-point (or 1.5-fold) difference between the municipalities with high (58.8%) and low (39.6%) happiness scores of 8 or higher. Additionally, we found a 22.6%-point (or 1.4-fold) gap between the municipalities with a score of 7 or higher and a 20.5%-point (or 2.6-fold) gap between the municipalities with a score of 5 or less. The pooled data and each survey year showed a large regional disparity in the aggregate score of 5 or fewer points for happiness.

The participating municipalities in this study had widely different social backgrounds and sociodemographic characteristics. For example, in terms of hobbies, there was a difference of 38.1%-point (or 4.1-fold) between the municipalities with the highest percentage of participation (50.4%) and those with the lowest (12.3%). Furthermore, there was a 51.7%-point (or 3.1-fold) gap between municipalities with low income, a 61.4%-point (or 14.3-fold) gap between the municipalities with low education, and a 29.3%-point (or 5.2-fold) gap between the municipalities with a percentage of individuals living alone.

Table 2 presents the results of the linear mixed-effects model evaluation regarding the relationship between social participation and happiness with a score of 8 or higher at the municipal level. Significant associations were found for six of the seven indicators of social participation, excluding neighborhood participation, indicating that the higher the percentage of social participation, the higher the percentage of the happiness score (8 or higher). This result was the same for all of the models. For example, in Model 2, a 10% higher participation in terms of skills was associated with a 3% higher happiness score (8 or higher).

Supplementary Tables S1 and S2 show the results of the sensitivity analyses wherein the cutoff points for happiness were set to 7 or more and 5 or fewer points, respectively. Even in the analysis in which the dependent variable had a score of 7 or higher for happiness, significant associations were found for six of the seven indicators of social participation, excluding neighborhood participation, indicating that the higher the percentage of social participation, the higher the percentage of happiness. However, in Model 1, a higher percentage of participation in the neighborhood category was associated with a lower percentage of happiness score (7 or higher). In the analysis where the dependent variable had 5 or fewer points, Models 1 and 2 showed significant associations for six of the seven indicators of social participation, excluding neighborhood participation, indicating that the higher the percentage of social participation, the lower the percentage of unhappiness. Moreover, in Model 1, a higher percentage of participation in the neighborhood category was associated with a higher percentage of happiness, with a score of 5 or lower.

## 4. Discussion

We examined the relationship between social participation by type and happiness at the municipal level by investigating community diagnostic indicators to determine the progress of building AFCs. Using linear mixed-effects models with pooled data from three waves of the JAGES, we found that the higher the percentage of social participation, the higher the percentage of happiness. Additionally, we used data from a large number of municipalities with various characteristics (e.g., rural to metropolitan areas) and adjusted for potential confounding factors. Although we tallied happiness using several cutoff points, our results were consistent.

According to the World Happiness Report [64], Japan’s average score for happiness from 2017 to 2019 was 5.871, or 62nd out of 153 countries. There were disparities among the municipalities for all cutoff points in the age-adjusted, municipal-level aggregated values of happiness. Among them, the difference and ratio between the maximum and minimum municipalities with less than five points below the average level of happiness in Japan were larger than the other cutoffs. Such disparities in terms of happiness in cutoff values below the mean indicate the need for improvement through regional development based on the AFC concept [3,16].

In this study, we used happiness as a measure of well-being; nonetheless, what constitutes a measure of well-being requires further discussion. The AFC concept does not mention what specific indicators of well-being should be used [3,16]. Thus, we focused on the availability indicator of happiness, a type of psychological well-being that is a basic item in the existing the Survey of Needs [37]. In community surveys and cross-cultural comparisons, using this single question to measure happiness has been shown to be reliable, valid, and feasible [39,40]. Reliability, validity, and feasibility are important properties required for community diagnostic indicators in AFCs. The AFC concept defines “impact” as a long-term change in people’s health (i.e., physical, cognitive, and emotional functioning) and well-being [16]. Happiness can be used as a variable depending on behavior [65], and intervention potential has also been reported [30,32,66]. Currently, the main age-friendly policies in Japan aim to promote the social participation of older people [22,23]. In the future, with the promotion of social participation of older adults, happiness as an impact indicator of AFC may also increase. Conversely, the happiness variable used in this study is an affective evaluation of the degree of intensity and content of happy moments and experiences in a person’s life [49,50]. Considering the long-term changes in the AFC concept [16], future research may need to use a different process to determine the quality of life, based on individual-specific criteria such as life satisfaction [48,49]. Furthermore, there is a need to develop a wide range of survey items and conduct research using those items in response to the expanding concepts of health and well-being [35,45,67].

The AFCCQ [4,6], a comprehensive 23-item questionnaire, is known as an existing community diagnostic indicator used to manage progress in building AFCs. The AFCCQ was developed through step-by-step, rigorous validation; it has been reported to be valid, psychometrically sound, and transparent [4,6]. It includes four questions in the domain of social participation: opportunities to meet in the neighborhood, accessibility of activities (events), information about activities (events), and abundance of activities (events) [6]. In this study, we used seven types of social participation that were commonly included in all three waves. According to the AFC concept [3,16], social participation generally includes leisure time participation in formal/informal religious, cultural, and other social activities with friends, relatives, and neighbors, especially in face-to-face encounters. The specific types of activities and frequency of participation can be determined by the community in which they occur [3,16]. Note that our study understands study social participation as an organized and regular activity and captures only one aspect of the social participation domain in the AFCCQ [4,6] and AFC concepts [3,16].

Regarding the association between social participation by type and happiness at the municipal level, it was similar for almost all types except for neighborhood participation. This result is consistent with several previous studies [4,10,11,14,34,35] that examined disparities and their associations with social participation and well-being at the national, urban, and community levels. However, only participation in the neighborhood had a significant negative or null correlation with happiness. Social activities of older adults were classified based on the degree of proximity to others and the goals of the activity [68,69]. Following this classification [68,69], the seven types of social participation used in this study can be divided into two main categories. Sports, hobbies, seniors, and learning can be categorized as task-oriented social activities that involve cooperation with others toward a common goal [68,69]. Volunteering, neighborhood activities, and skills development can be classified as activities oriented toward helping others, which means helping a specific person or group of people [68,69]. Happiness is more closely related to good social relationships and health behaviors, and social factors such as positive social support are important for predicting increased happiness [65,66]. Although this study cannot address the temporal pre- and post-relationships, four of the task-oriented organizations and two that are oriented toward helping others were positively associated with happiness at the municipal level. “The dark side of social capital” [70] may have been related to the fact that among the activity associations oriented toward helping others, only neighborhood associations showed a negative association with happiness. Thus, a neighborhood is considered to be a vertical organization with clear hierarchical structures and relationships [71,72]. Because vertical organizational structures can become obligatory with an increase in participation frequency, they can be subject to what researchers call “the dark side of social capital” [70]. In this regard, self-rated health and physical and mental health can deteriorate with frequent and obligatory rather than voluntary social participation [73,74]. This “dark side” may have emerged in the relationship between neighborhood and happiness.

Finally, this study has some limitations. First, because this study is a cross-sectional analysis that pooled data from three waves of the JAGES, it is not possible to comment on the causal relationship between social participation and happiness at the municipal level. However, this study did not aim to make causal inferences but examined community diagnostic indicators that are useful for AFC research and building. Either direction of the causal relationship between social participation and happiness would be a useful community diagnosis indicator. Second, as this is an ecological study, it is not possible to distinguish between compositional and contextual effects. However, ecological indicators and concepts that reflect compositional and contextual effects are considered to be important in AFC building. Third, this is a single country study and we used seven types of social participation, which may not capture interactions with other age groups but only as a portion of AFC’s social participation domain. Finally, there is a possibility of selection bias. It has been reported that low-income older people are less likely to respond to surveys [75]. Therefore, the results of this study are likely to be underestimated because non-responding people may not want to participate and reveal their poor well-being.

## 5. Conclusions

In this study, we examined the relationship between social participation by type and happiness at the municipal level to examine community diagnostic indicators to manage progress in building AFCs. We used three-wave data from the JAGES that had widely different social backgrounds and sociodemographic characteristics. The results showed that even after controlling for potential confounders, municipalities with a higher percentage of social participation, except for neighborhood participation, showed higher happiness. By making it easier for older adults to participate in society, we have the potential to create an AFC—a city full of happy older adults. Our study suggests that social participation is useful as a community diagnostic indicator for monitoring the progress in building AFCs, developing strategies, and creating evidence. The study had the limitation that it was single country study of only seven social participation types, and other types of social participation were not considered. Further research will be needed to confirm the transferability of the finding of this study to the type of social participation in other countries.

## Figures and Tables

**Table 1:** Age-adjusted descriptive statistics of municipal factors.

Variables	All (*n* = 289)				2013 (*n* = 77)				2016 (*n* = 91)				2019 (*n* = 121)			
	Mean	SD	Min	Max	Mean	SD	Min	Max	Mean	SD	Min	Max	Mean	SD	Min	Max
Happiness ≥ 8 points (%)	51.4	3.7	39.6	58.9	53.0	3.4	43.8	58.9	51.6	3.2	42.3	57.8	50.3	3.8	39.6	58.8
Happiness ≥ 7 points (%)	69.1	4.0	55.6	79.1	69.8	3.9	57.8	79.1	68.8	3.4	60.8	76.2	68.9	4.4	55.6	78.2
Happiness ≤ 5 points (%)	20.2	3.7	8.9	33.3	18.2	3.5	8.9	31.9	21.3	3.0	14.9	28.2	20.7	3.9	12.8	33.3
Volunteering (%)	14.2	2.8	6.6	25.2	12.4	2.3	8.2	18.4	15.6	2.4	6.6	25.2	14.4	2.7	6.9	22.5
Sports (%)	27.6	5.9	6.6	40.8	24.8	5.6	6.6	38.7	29.7	5.2	13.8	40.8	27.9	5.8	7.2	40.7
Hobbies (%)	35.3	6.1	12.3	50.4	33.7	5.4	12.3	44.6	38.5	5.6	21.1	50.4	34.0	6.1	13.7	43.8
Seniors (%)	8.7	3.6	1.7	22.5	9.0	3.7	4.5	19.0	9.1	3.4	4.3	18.7	8.1	3.6	1.7	22.5
Neighborhood (%)	10.8	3.2	3.3	24.1	10.4	3.1	4.8	18.7	10.6	2.9	3.3	19.1	11.2	3.4	4.3	24.1
Learning (%)	10.1	2.7	3.0	17.4	9.9	2.6	3.0	16.7	10.5	2.8	3.8	15.9	10.0	2.8	3.0	17.4
Skills (%)	6.8	1.8	2.1	13.1	6.2	1.5	2.4	10.5	7.5	1.7	4.0	12.2	6.5	1.8	2.1	13.1
Low income (%)	47.9	9.3	24.7	76.4	49.5	9.4	29.0	76.4	48.9	8.6	27.9	73.2	46.2	9.6	24.7	70.6
Low education (%)	29.8	13.3	4.6	66.0	37.2	13.4	14.2	66.0	31.0	12.2	12.0	61.4	24.2	11.5	4.6	65.2
Living alone (%)	16.9	5.3	7.0	36.3	16.4	5.5	7.0	31.2	17.8	5.6	8.4	36.3	16.6	4.9	7.9	35.1
Self-reported medical illness (%)	81.0	2.8	74.5	88.0	83.8	2.1	78.1	88.0	79.8	2.1	74.5	84.3	80.2	2.5	75.1	85.6
Poor self-rated health (%)	12.9	3.1	6.5	22.4	16.6	2.4	11.9	22.4	12.2	1.9	8.6	17.1	11.1	2.0	6.5	16.3
Depression ^1^ (%)	22.4	3.6	15.3	37.0	25.6	3.3	19.7	37.0	21.2	2.7	16.4	29.8	21.4	3.2	15.3	31.3
Population density ^2^	*n*		%		*n*		%		*n*		%		*n*		%	
Metropolitan	166		57.4		40		51.9		50		54.9		76		62.8	
Urban	56		19.4		24		31.2		18		19.8		14		11.6	
Rural	67		23.2		13		16.9		23		25.3		31		25.6	

SD: Standard deviation. Min: Minimum. Max: Maximum. All of the factors were adjusted for age using the direct methods. ^1^ Depression was defined according to the score of ≥5 points on the Geriatrics Depression Scale. ^2^ Population density of habitable land: Metropolitan (≥4000 people/km^2^), Urban (1000–3999 people/km^2^), or Rural (<1000 people/km^2^).

**Table 2:** The results of the linear mixed-effects model evaluation of the relationship between social participation and happiness with a score of 8 or higher at the municipal level.

Variables	Crude				Model 1				Model 2			
	*B*	95% CI		*p*	*B*	95% CI		*p*	*B*	95% CI		*p*
Volunteering (%)	0.49	0.33	0.64	<0.001	0.28	0.13	0.43	<0.001	0.16	0.03	0.29	0.015
Sports (%)	0.33	0.27	0.39	<0.001	0.30	0.21	0.39	<0.001	0.15	0.06	0.23	0.001
Hobbies (%)	0.32	0.26	0.38	<0.001	0.31	0.21	0.41	<0.001	0.14	0.05	0.23	0.004
Seniors (%)	0.12	0.01	0.23	0.040	0.33	0.22	0.44	<0.001	0.21	0.12	0.31	<0.001
Neighborhood (%)	−0.09	−0.22	0.04	0.160	−0.09	−0.20	0.02	0.114	−0.05	−0.14	0.04	0.276
Learning (%)	0.51	0.38	0.65	<0.001	0.43	0.23	0.62	<0.001	0.20	0.03	0.37	0.024
Skills (%)	0.86	0.64	1.08	<0.001	0.54	0.27	0.82	<0.001	0.30	0.06	0.54	0.014

*B*: Unstandardized coefficients. CI: Confidence interval. All of the factors were adjusted for age using the direct methods. The dependent variable was happiness with a score of 8 points or higher. The data consisted in an iterative cross-sectional design that had two levels, i.e., Level 1 municipal level aggregates were nested within Level 2 survey years. Model 1: Crude model and low equivalent income, low education, living alone at the municipal level, and population density of habitable land. Model 2: Model 1 and self-reported medical illness, poor self-rated health, and depression.

## Data Availability

The data were from the JAGES. For inquiries, please contact the Data Management Committee (e-mail: dataadmin.ml@jages.net). All of the JAGES datasets contain confidential subject information and therefore have ethical or legal restrictions on release. The JAGES Data Management Committee has established these restrictions, with the guidance of the local authorities who contributed to the survey. Researchers will be able to use the data once they submit a research plan (using the JAGES data) and have it approved by the registered researchers.

## Supplementary Materials

The following supporting information can be downloaded at: https://www.mdpi.com/article/10.3390/ijerph19095096/s1, Table S1: The results of the linear mixed-effects model evaluation of the relationship between social participation and happiness with a score of 7 or higher at the municipal level; Table S2: The results of the linear mixed-effects model evaluation of the relationship between social participation and happiness with a score of less than 5 at the municipal level.
