# Supplementary material for: Suggesting Indicators of Age-Friendly City: Social Participation and Happiness, an Ecological Study from the JAGES
Source: Int J Environ Res Public Health. 2022 Apr 22;19(9):5096. doi: 10.3390/ijerph19095096 (PMC9102036; doi:10.3390/ijerph19095096)
Supplement: Supplementary file 1 [file ijerph-19-05096-s001.zip › ijerph-1606202-SI.pdf]

International Journal of
Environmental Research and Public Health

MDPI

Supplementary Materials

Table S1. The results of the linear mixed-effects model evaluation of the relationship between social participation and happiness scores of 7 or higher at the municipal level.

Variables	Crude				Model 1				Model 2			
	B	95% CI		*p*	*B*	95% CI		*p*	*B*	95% CI		*p*
Volunteering (%)	0.65	0.49	0.82	< 0.001	0.24	0.10	0.38	< 0.001	0.11	0.00	0.23	0.052
Sports (%)	0.50	0.44	0.56	< 0.001	0.31	0.23	0.39	< 0.001	0.16	0.09	0.24	< 0.001
Hobbies (%)	0.51	0.45	0.56	< 0.001	0.36	0.27	0.45	< 0.001	0.20	0.12	0.28	< 0.001
Seniors (%)	−0.04	−0.17	0.09	0.567	0.32	0.22	0.42	< 0.001	0.20	0.12	0.28	< 0.001
Neighborhood (%)	−0.06	−0.20	0.08	0.411	−0.11	−0.21	−0.01	0.029	−0.07	−0.16	0.01	0.072
Learning (%)	0.82	0.68	0.96	< 0.001	0.42	0.25	0.60	< 0.001	0.20	0.05	0.35	0.011
Skills (%)	1.31	1.09	1.54	< 0.001	0.50	0.24	0.75	< 0.001	0.25	0.04	0.46	0.020

B: Unstandardized coefficients. CI: Confidence interval. All of the factors were adjusted for age using the direct methods. The dependent variable was happiness with a score of 7 points or higher. The data was an iterative cross-sectional design that consisted of two levels; i.e., Level 1 municipal level aggregates were nested within Level 2 survey years. Model 1: Crude model + low equivalent income, low education, living alone at the municipal level, and population density of habitable land. Model 2: Model 1 + self-reported medical illness, poor self-rated health, and depression.

Table S2. The results of the linear mixed-effects model evaluation of the relationship between social participation and happiness scores of less than 5 at the municipal level.

Variables	Crude				Model 1				Model 2			
	B	95% CI		p	B	95% CI		p	B	95% CI		p
Volunteering (%)	−0.63	−0.77	−0.48	< 0.001	−0.23	−0.34	−0.11	< 0.001	−0.13	−0.23	−0.03	0.011
Sports (%)	−0.47	−0.52	−0.42	< 0.001	−0.29	−0.35	−0.22	< 0.001	−0.18	−0.24	−0.11	< 0.001
Hobbies (%)	−0.48	−0.52	−0.43	< 0.001	−0.34	−0.41	−0.27	< 0.001	−0.22	−0.29	−0.15	< 0.001
Seniors (%)	0.09	−0.03	0.20	0.139	−0.24	−0.32	−0.16	< 0.001	−0.14	−0.22	−0.07	< 0.001
Neighborhood (%)	0.03	−0.10	0.15	0.697	0.09	0.00	0.17	0.045	0.06	−0.01	0.13	0.117
Learning (%)	−0.76	−0.88	−0.64	< 0.001	−0.35	−0.49	−0.20	< 0.001	−0.16	−0.29	−0.03	0.016
Skills (%)	−1.23	−1.43	−1.04	< 0.001	−0.43	−0.65	−0.22	< 0.001	−0.24	−0.42	−0.06	0.010

B: Unstandardized coefficients. CI: Confidence interval. All of the factors were adjusted for age using the direct methods. The dependent variable was happiness with a score of 5 points or less. The data was an iterative cross-sectional design that consisted of two levels; i.e., Level 1 municipal level aggregates were nested within Level 2 survey years. Model 1: Crude model + low equivalent income, low education, living alone at the municipal level, and population density of habitable land. Model 2: Model 1 + self-reported medical illness, poor self-rated health, and depression.
